# Supplementary material for: A Global Screen for Assembly State Changes of the Mitotic Proteome by SEC-SWATH-MS
Source: Cell Syst. 2020 Feb 26;10(2):133–155.e6. doi: 10.1016/j.cels.2020.01.001 (PMC7042714; doi:10.1016/j.cels.2020.01.001)

Q9BYX2 | TBD2A_HUMAN | TBC1D2 PARIS1 PP8997 TBC1D2A
Monomer MW [kDa]: 105.414 Monomer expected elution fraction: 40

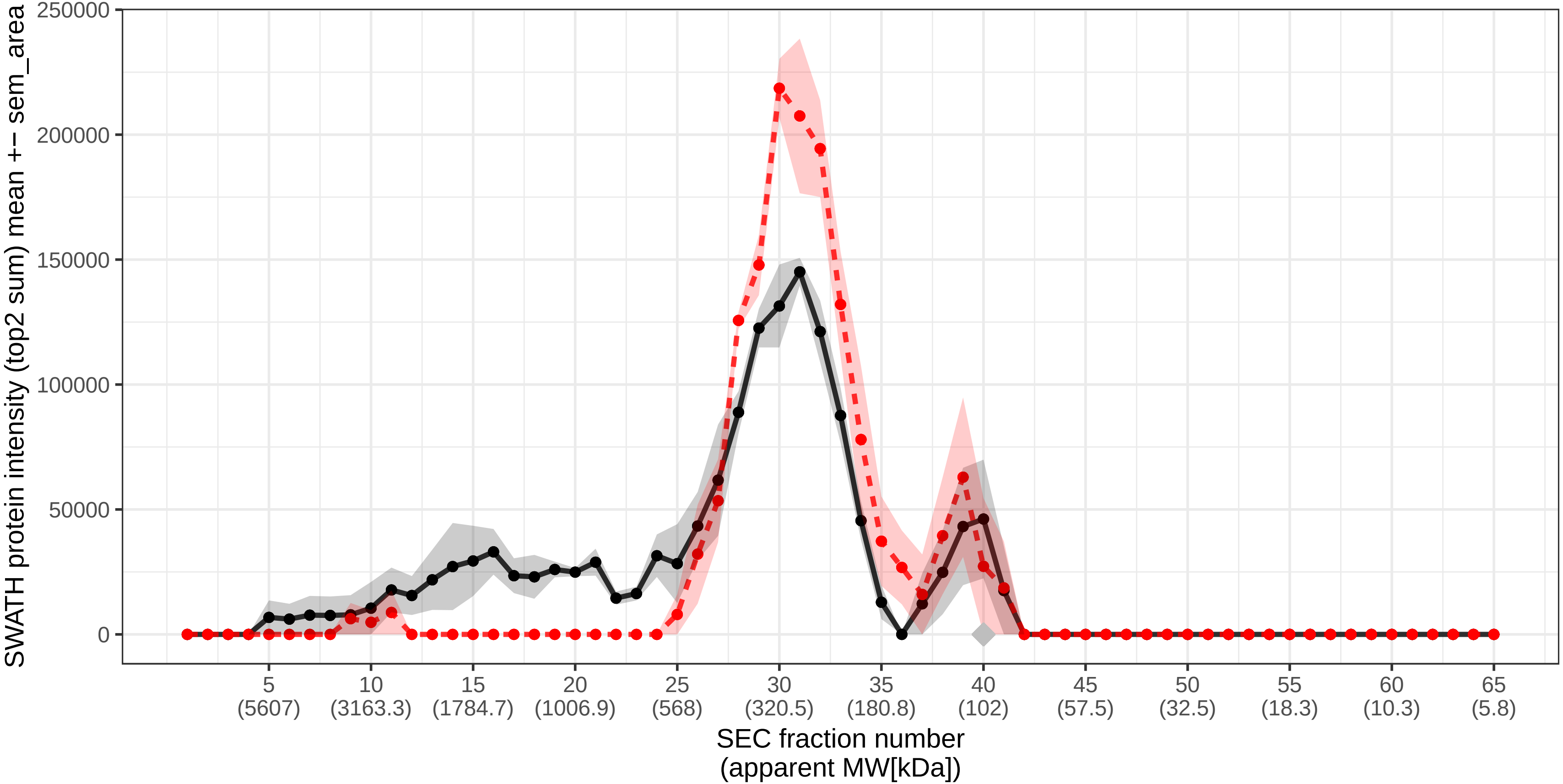

Supplement: Data S1. SEC-SWATH-MS Protein Chromatograms, Related to Figure 1 [file mmc6.zip › SECchrom_Q9BYX2_TBD2A_HUMAN_TBC1D2_PARIS1_PP8997_TBC1D2A.pdf]
